# Exploring the mechanism of *Ginkgo biloba* L. leaves in the treatment of vascular dementia based on network pharmacology, molecular docking, and molecular dynamics simulation

**DOI:** 10.1097/MD.0000000000033877

**Published:** 2023-05-26

**Authors:** Jienuo Pan, Jiqin Tang, Jialin Gai, Yilan Jin, Bingshun Tang, Xiaohua Fan

**Affiliations:** a School of Rehabilitation Medicine, Shandong University of Traditional Chinese Medicine, Jinan, China; b School of International Education, Shandong University of Traditional Chinese Medicine, Jinan, China; c School of Traditional Chinese Medicine, Shandong University of Traditional Chinese Medicine, Jinan, China; d Department of Rehabilitation Medicine, Provincial Hospital Affiliated to Shandong First Medical University, Jinan, China.

**Keywords:** *Ginkgo biloba* L. leaves, molecular docking, molecular dynamics simulation, network pharmacology, vascular dementia

## Abstract

*Ginkgo biloba* L. leaves (GBLs) play a substantial role in the treatment of vascular dementia (VD); however, the underlying mechanisms of action are unclear.

**Objective:**

This study was conducted to investigate the mechanisms of action of GBLs in the treatment of VD through network pharmacology, molecular docking, and molecular dynamics simulations.

**Methods:**

The active ingredients and related targets of GBLs were screened using the traditional Chinese medicine systems pharmacology, Swiss Target Prediction and GeneCards databases, and the VD-related targets were screened using the OMIM, DrugBank, GeneCards, and DisGeNET databases, and the potential targets were identified using a Venn diagram. We used Cytoscape 3.8.0 software and the STRING platform to construct traditional Chinese medicine–active ingredient–potential target and protein–protein interaction networks, respectively. After gene ontology and Kyoto Encyclopedia of Genes and Genomes pathway analysis of potential targets using the DAVID platform, the binding affinity between key active ingredients and targets was analyzed by molecular docking, and finally, the top 3 proteins–ligand pairs with the best binding were simulated by molecular dynamics to verify the molecular docking results.

**Results:**

A total of 27 active ingredients of GBLs were screened and 274 potential targets involved in the treatment of VD were identified. Quercetin, luteolin, kaempferol, and ginkgolide B were the core ingredients for treatment, and AKT1, TNF, IL6, VEGFA, IL1B, TP53, CASP3, SRC, EGFR, JUN, and EGFR were the main targets of action. The main biological processes involved apoptosis, inflammatory response, cell migration, lipopolysaccharide response, hypoxia response, and aging. PI3K/Akt appeared to be a key signaling pathway for GBLs in the treatment of VD. Molecular docking displayed strong binding affinity between the active ingredients and the targets. Molecular dynamics simulation results further verified the stability of their interactions.

**Conclusion subsections:**

This study revealed the potential molecular mechanisms involved in the treatment of VD by GBLs using multi-ingredient, multi-target, and multi-pathway interactions, providing a theoretical basis for the clinical treatment and lead drug development of VD.

## 1. Introduction

Vascular dementia (VD) is a neurocognitive disorder caused by the global or local impact of vascular diseases that leads to stroke injury or other changes in tissue perfusion and is characterized by behavioral abnormalities, motor and autonomic dysfunction, and neurocognitive impairment.^[1]^ It is often considered one of the most common causes of dementia after Alzheimer’s disease, accounting for approximately 15% to 20% of dementia cases in Western developed countries and possibly up to 30% in Asia and developing countries,^[2,3]^ with a median survival time of approximately 4 to 9.4 years.^[4,5]^ Cerebrovascular factors play a key role in the pathogenesis of VD and cerebrovascular diseases such as chronic cerebral hypoperfusion (CCH), cerebral small vessel lesions, cerebral amyloid angiopathy, and mixed vascular lesions can trigger the molecular mechanisms leading to VD.^[1,6]^ Among these conditions, CCH is considered a key etiological factor in the progression of VD.^[7]^ However, the specific pathogenesis of VD remains unclear; therefore, its management is highly limited and there are currently no approved standard VD treatments to date. Cholinesterase inhibitors and memantine have been found to show some cognitive improvement, but the effect is very limited and expensive, and the price of weak efficacy increases the risk of adverse events in patients.^[8,9]^ The ideal drug for VD should not only improve cognitive impairment but also have multiple other effects, including angiogenesis and neuroprotection. The unique advantages of multi-component, multi-target, and multi-pathway interactions of traditional Chinese medicine (TCM) provide new ideas for the development of ideal drugs for VD. Numerous studies have shown that TCM have clinical effects on VD, and compared to singular drugs, they have much more complicated targets and mechanisms. Compared to current prescription drugs, TCM exhibits fewer adverse effects, lower costs, and higher applicability for long-term use and has considerable potential in the treatment of VD.^[10]^

**Figure 1.**
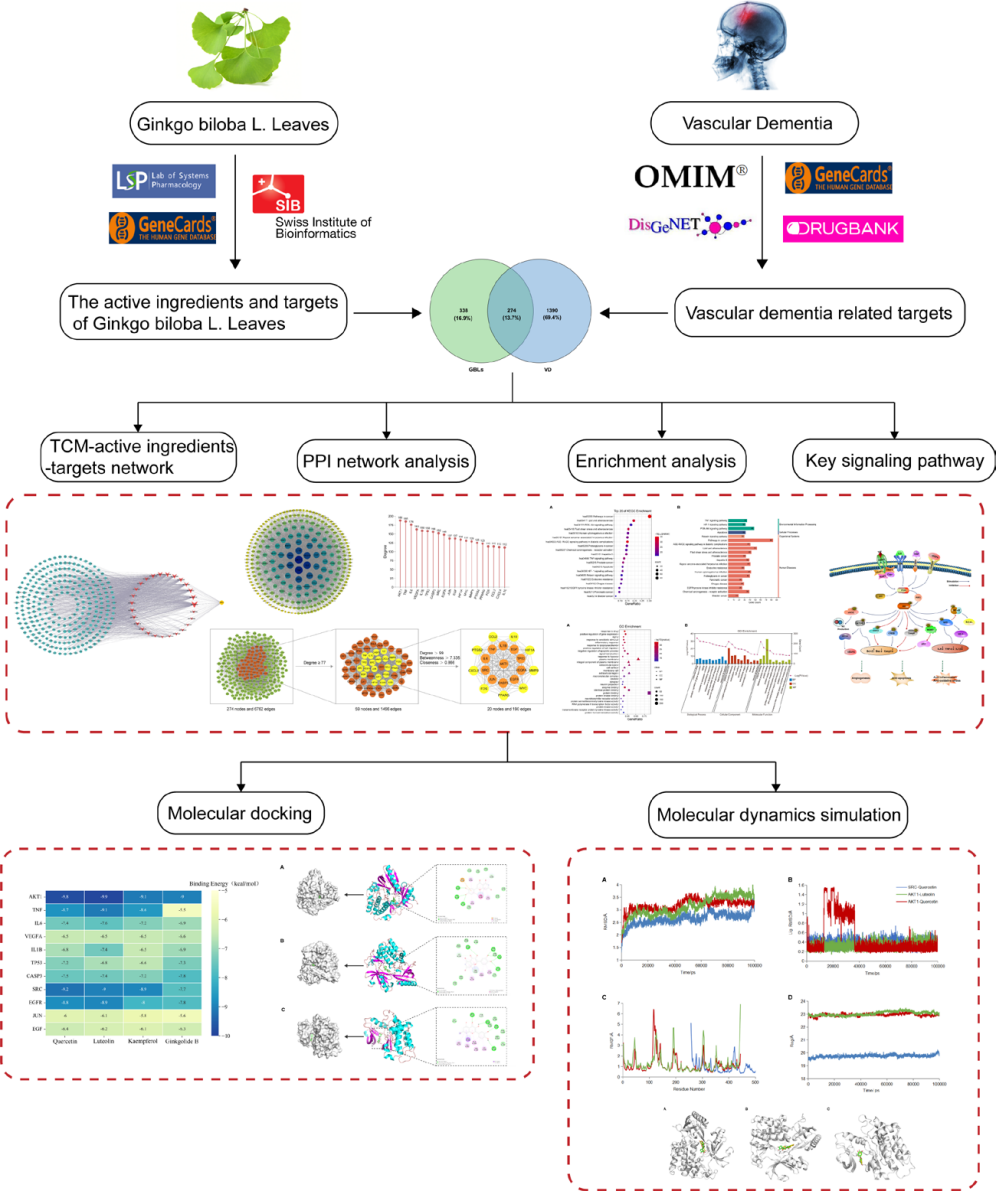
The flow chart of this study. OMIM = Online Mendelian Inheritance in Man, PPI = protein–protein interactional, TCM = traditional Chinese medicine.

VD belongs to the category of “dementia” and “forgetfulness” in Chinese medicine. Modern Chinese physicians have different opinions on the pathogenesis of VD, but most revolve around the theoretical system of deficiency in the spleen and kidney is deficiency and an excess in the combination of phlegm and blood stasis. Therefore, the primary clinical treatment is to invigorate the kidney and spleen, benefit qi and remove blood stasis, promote blood circulation and open the orifices, and dissipate phlegm for resuscitation.^[11]^ *Ginkgo biloba* L. leaves (GBLs) are a commonly used TCM for treating cardiovascular and cerebrovascular diseases. They are rich in various natural active ingredients with a wide range of pharmacological activities, playing an important role in food, health care and medicine and have a high medicinal value. The Chinese Pharmacopoeia (2020 edition) records that GBLs have the effects of promoting blood circulation for removing blood stasis, regulating collaterals for relieving pain, removing turbidity and lowering lipids. Therefore, it is mainly used clinically to treat patients with dementia, memory decline, ischemic stroke, coronary angina and other brain dysfunction, degenerative diseases, and peripheral and coronary vascular diseases.^[12]^ Western medical research has found that GBLs have antioxidant, anti-inflammatory, anti-apoptosis, enhanced neuroplasticity, reduced blood viscosity, and enhanced microperfusion activity; show good safety and tolerance; and are increasingly used for the treatment of cognitive impairment with or without cerebrovascular disease because of their known neuroprotective effects and cerebrovascular benefits.^[13]^ *Ginkgo biloba* extract (GBE) is derived from GBLs and is used as a nutritional supplement and drug to improve cardiovascular and cerebrovascular diseases in many countries. The Anatomical Therapeutic Chemical classification system classifies GBE as an antidementia drug, along with acetylcholinesterase inhibitors (AChEIs) and memantine. The expert consensus in recent years states that the use of GBE alone or as an additional therapy plays an important role in the treatment of dementia, especially when patients have poor efficacy with AChEIs or memantine.^[14]^ Although GBLs are widely used to treat dementia, systematic studies on the direct effects and mechanisms of GBLs on VD are still lacking. The current study, therefore, used network pharmacology combined with molecular docking and molecular dynamics simulations to investigate and predict the underlying mechanisms of GBLs for the treatment of VD and provides ideas for clinical applications and experimental studies of GBLs and VD (Fig. 1).

## 2. Materials and methods

### 2.1. Screening of active ingredients and target genes of GBLs

All active ingredients of GBLs were searched through the Traditional Chinese Medicine System Pharmacology Database and Analysis Platform (TCMSP) database^[15]^ (https://old.tcmsp-e.com/tcmsp.php) . All ingredients were screened, and their targets were recorded with oral bioavailability ≥ 30% and drug-likeness ≥ 0.18 as the qualifying conditions. The normalized SMILES structures of each active ingredient were retrieved from the PubChem database^[16]^ (https://pubchem.ncbi.nlm.nih.gov/) and entered into the Swiss Target Prediction Database^[17]^ (http://www.swisstargetprediction.ch). Target prediction was performed using the species “*Homo sapiens*” and a probability > 0.1 as the screening criteria. The GeneCards human gene database^[18]^ (http://www.genecard.org/) was used to collect unpredicted active ingredient targets. Finally, the target proteins were converted and normalized using the UniProt database^[19]^ (http://www.uniprot.org) and the targets of GBLs were integrated and de-duplicated.

### 2.2. VD disease-related target prediction

Using “Vascular dementia” as the keyword, the disease targets were searched and screened in OMIM^[20]^ (http://omim.org/), DrugBank^[21]^ (http://www.drugbank.ca), DisGeNET^[22]^ (https://www.disgenet.org/), and GeneCards databases for disease target search and screening, integrated, and de-duplicated to obtain VD disease targets.

### 2.3. Constructing a “Traditional Chinese Medicine (TCM)–active ingredients–potential targets” network

The Venn diagram was constructed to obtain the intersection of GBLs and VD disease targets, i.e., the potential targets of GBLs for VD treatment. Cytoscape 3.8.0 software^[23]^ was used to construct the “TCM***–***active ingredients–potential targets” network. The nodes in the network diagram represent the drugs, active ingredients, and potential targets and the edges represent the interactions between the 3. The topological properties of the network were analyzed using the Network Analyzer tool in Cytoscape and the 3 parameters of degree, betweenness centrality, and closeness centrality were used to analyze the key active ingredients of GBLs in treating VD. The node size reflects the degree value.

### 2.4. Protein–protein interaction (PPI) network construction and key target screening

To identify the interaction targets of GBLs that may be relevant to VD pathology and treatment, the potential targets were uploaded to the STRING database^[24]^ (https://string-db.org) to construct a PPI network for the species *Homo sapiens*, with a confidence level of 0.4. Default settings were used for the other parameters and the PPI network of potential targets was obtained. The network files were imported into Cytoscape to visualize and analyze the topological properties of the PPI network; the target color and size reflected the node degree value. The first screening was qualified with greater than or equal to twice the median degree value; the second screening was subsequently performed to identify targets with greater than the median degree, median betweenness, and median closeness, i.e., key targets.

### 2.5. Gene ontology (GO) and Kyoto Encyclopedia of Genes and Genomes (KEGG) pathway enrichment analysis

To further investigate the underlying mechanisms of GBLs for VD treatment, GO functional enrichment analysis (including biological processes, molecular functions, and cellular components) and KEGG pathway enrichment analysis (*P* < .05) were performed using the DAVID database^[25]^ (https://david.ncifcrf.gov) for potential targets with the species limited to *Homo sapiens*. The top 10 GO entries and top 20 KEGG signaling pathways were screened based on their *P* values and a bubble diagram and histograms were generated using Microscopic Letters (http://www.bioinformatics.com.cn) and GraphPad Prism.

### 2.6. Molecular docking and visualization

The top 11 key targets in the degree ranking were subjected to molecular docking analysis with the 4 key active ingredients and the docking binding energy was calculated. The three-dimensional (3D) structures of the key active ingredients were downloaded from the PubChem database and the 3D stereo structures of the core target proteins were obtained from the PDB database (https://www.rcsb.org/). The target proteins and small molecules were processed using AutoDockTool 1.5.6 software to remove water, add hydrogen, add charge, and set rotatable bonds. Then, all processed small molecules, as well as proteins, were converted to PDBQT format. Finally, semi-flexible molecular docking was performed using AutoDock Vina 1.1.2 and the docking results were visualized using Discovery Studio 4.5 and PyMol 2.5.2 software to obtain 2D and 3D images.^[26]^

### 2.7. Molecular dynamics simulations

The binding affinity of the key active ingredients after docking with the target proteins was further analyzed using molecular dynamics simulations. Molecular dynamics simulations were performed using Amber18 software^[27]^ for the 3 groups of complexes which displayed high binding affinity during molecular docking. The ff14SB force field parameters were used for proteins and gaff2 generic force field parameters were used for active ingredients. The selected TIP3P explicit water model and periodic boundary conditions were set. Energy optimization, heating, and equilibration were performed for all systems. Finally, the systems were subjected to molecular dynamics simulations for 100 ns under the npt ensemble with a time step of 2 fs and the trajectory data were saved every 20 ps and analyzed using the CPPTRAJ module. Binding energy calculations for the active ingredients and proteins were performed using the MMPBSA.py module.

## 3. Results

### 3.1. Active ingredients and potential targets of GBLs

After searching the TCMSP database, a total of 27 major active ingredients of GBLs that met the conditions were obtained (Table 1). A total of 229, 407, and 95 potential targets were obtained from TCMSP, Swiss Target Prediction, and GeneCards databases, respectively. After the targets were standardized and de-duplicated using the UniProt database, 612 possible action targets of GBLs were obtained.

**Table 1:** Active ingredients and ADME parameters of GBLs.

MOL ID	Name of ingredients	Formula	OB (%)	DL
MOL011578	Bilobalide	C_15_H_18_O_8_	84.42	0.36
MOL002680	Flavoxanthin	C_40_H_56_O_3_	60.41	0.56
MOL011586	Ginkgolide B	C_20_H_24_O_10_	44.38	0.73
MOL011587	Ginkgolide C	C_20_H_24_O_11_	48.33	0.73
MOL011588	Ginkgolide J	C_20_H_24_O_10_	44.84	0.74
MOL011589	Ginkgolide M	C_20_H_24_O_10_	49.09	0.75
MOL011594	Isogoycyrol	C_21_H_18_O_6_	40.36	0.83
MOL011597	Luteolin-4′-glucoside	C_21_H_20_O_11_	41.97	0.79
MOL011604	Syringetin	C_17_H_14_O_8_	36.82	0.37
MOL001490	Bis[(2S)-2-ethylhexyl] benzene-1,2-dicarboxylate	C_24_H_38_O_4_	43.59	0.35
MOL001494	Mandenol	C_20_H_36_O_2_	42.00	0.19
MOL001558	Sesamin	C_20_H_18_O_6_	56.55	0.83
MOL002881	Diosmetin	C_16_H_12_O_6_	31.14	0.27
MOL003044	Chryseriol	C_16_H_12_O_6_	35.85	0.27
MOL000354	Isorhamnetin	C_16_H_12_O_7_	49.60	0.31
MOL000358	Beta-sitosterol	C_29_H_50_O	36.91	0.75
MOL000422	Kaempferol	C_15_H_10_O_6_	41.88	0.24
MOL000449	Stigmasterol	C_29_H_48_O	43.83	0.76
MOL000492	(+)-Catechin	C_15_H_14_O_6_	54.83	0.24
MOL005573	Genkwanin	C_16_H_12_O_5_	37.13	0.24
MOL000006	Luteolin	C_15_H_10_O_6_	36.16	0.25
MOL007179	Linolenic acid ethyl ester	C_20_H_34_O_2_	46.10	0.20
MOL009278	Laricitrin	C_16_H_12_O_8_	35.38	0.34
MOL000096	(-)-Catechin	C_15_H_14_O_6_	49.68	0.24
MOL000098	Quercetin	C_15_H_10_O_7_	46.43	0.28
MOL002883	Ethyl oleate (NF)	C_20_H_38_O_2_	32.40	0.19
MOL005043	Campest-5-en-3beta-ol	C_28_H_48_O	37.58	0.71

ADME = absorption, distribution, metabolism, and excretion, DL = drug-likeness, GBLs = *Ginkgo biloba* L. leaves, OB = oral bioavailability.

### 3.2. VD disease-related targets

We obtained 1092, 622, 216, and 50 VD-related targets from the GeneCards (correlation score > 4.74), OMIM, DisGeNET, and DrugBank databases, respectively. A total of 1665 VD targets were obtained by collation and deduplication.

### 3.3. TCM–active ingredients–potential targets network analysis

**Figure 2.**
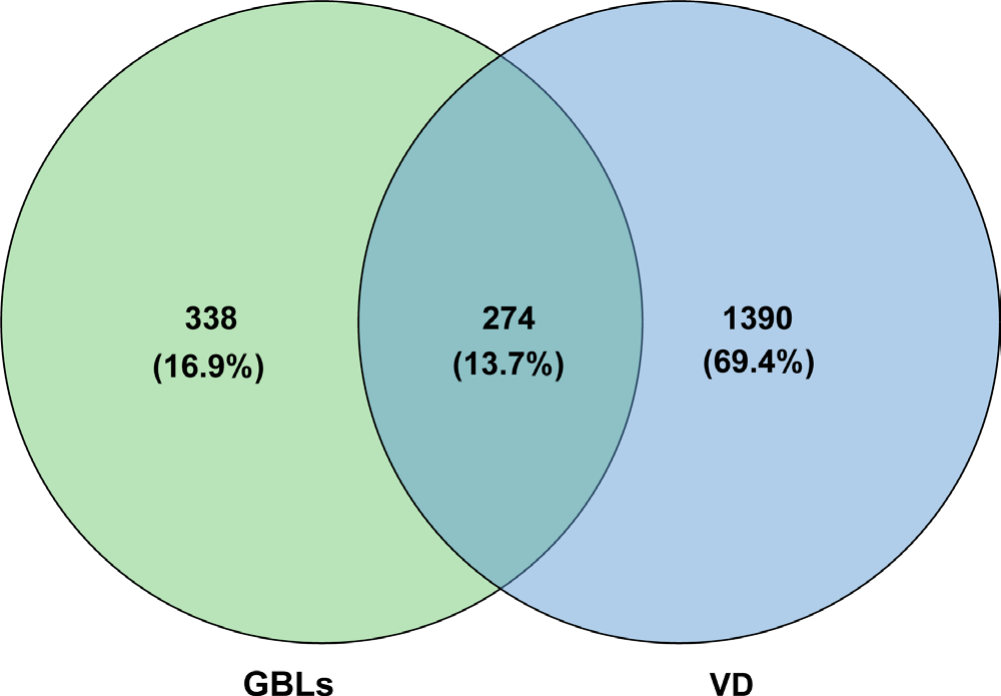
GBLs–VD intersection targets Venn diagram. GBLs = *Ginkgo biloba* L. leaves, VD = vascular dementia.

**Figure 3.**
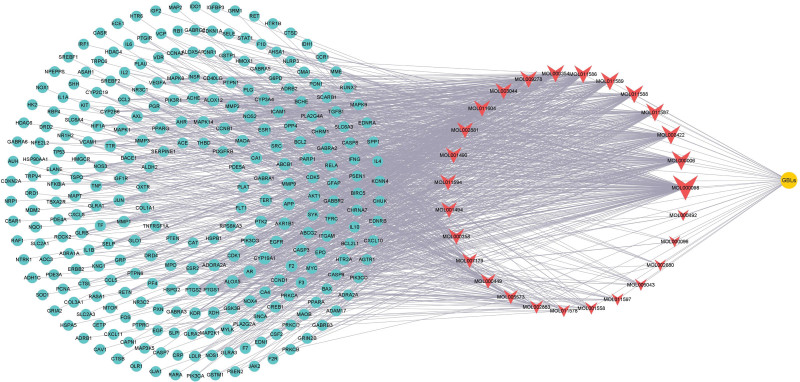
Traditional Chinese medicine-active ingredients-potential targets network (the orange circle represents traditional Chinese medicine, the red inverted triangle represents active ingredients, and the blue circle represents potential targets).

A Venn diagram was used to identify 274 intersectional targets of both GBLs and VD (Fig. 2). The active ingredients and potential targets of GBLs were imported into Cytoscape 3.8.0 software to construct a TCM***–***active ingredients–potential targets network, which included 302 nodes and 1203 pairs of interactions (Fig. 3). The results were analyzed using a Network Analyzer and indicated mean values of the network degree, betweenness centrality, and closeness centrality of 7.97, 0.0070, and 0.33, respectively. The top active ingredients were the flavonoids quercetin, luteolin, and kaempferol and the terpenoid ginkgolide. Among the ginkgolides, ginkgolide B has more potent activity, and therefore, has been the subject of more pharmacological research. Moreover, it may be an important active component of GBLs in the treatment of VD. The topological parameters are listed in Table 2.

**Table 2:** Topological analysis of GBLs-active ingredients-potential targets network.

Name of ingredients	Degree	Betweenness centrality	Closeness centrality
Quercetin	132	0.333742748	0.491027732
Luteolin	78	0.081698541	0.417475728
Kaempferol	75	0.061938416	0.414030261
Ginkgolide B/J/C/M	66	0.05311654	0.404026846
Isorhamnetin	61	0.038684471	0.398675497
Laricitrin	56	0.024251852	0.393464052
Chryseriol	50	0.01780968	0.387387387
Syringetin	50	0.015832806	0.387387387
Bis[(2S)-2-ethylhexyl] benzene-1,2-dicarboxylate	48	0.128991471	0.385403329
Diosmetin	48	0.016009138	0.385403329
Isogoycyrol	47	0.111044299	0.384418902
Mandenol	46	0.111464792	0.38343949
Beta-sitosterol	46	0.082579381	0.38343949
Linolenic acid ethyl ester	38	0.061160894	0.375780275
Stigmasterol	38	0.057207712	0.375780275
Genkwanin	33	0.008288899	0.371146732
Ethyl oleate (NF)	23	0.023142654	0.3622142
Sesamin	15	0.037018137	0.355371901
Bilobalide	15	0.016137695	0.355371901
Luteolin-4′-glucoside	12	0.015721211	0.352872216
Campest-5-en-3beta-ol	11	0.002890833	0.352046784
Flavoxanthin	7	0.001652343	0.348783314
(+)-Catechin	5	0.006709368	0.347174164
(-)-Catechin	5	0.000135	0.347174164

GBLs = *Ginkgo biloba* L. leaves.

### 3.4. PPI network analysis and key target screening results

**Figure 4.**
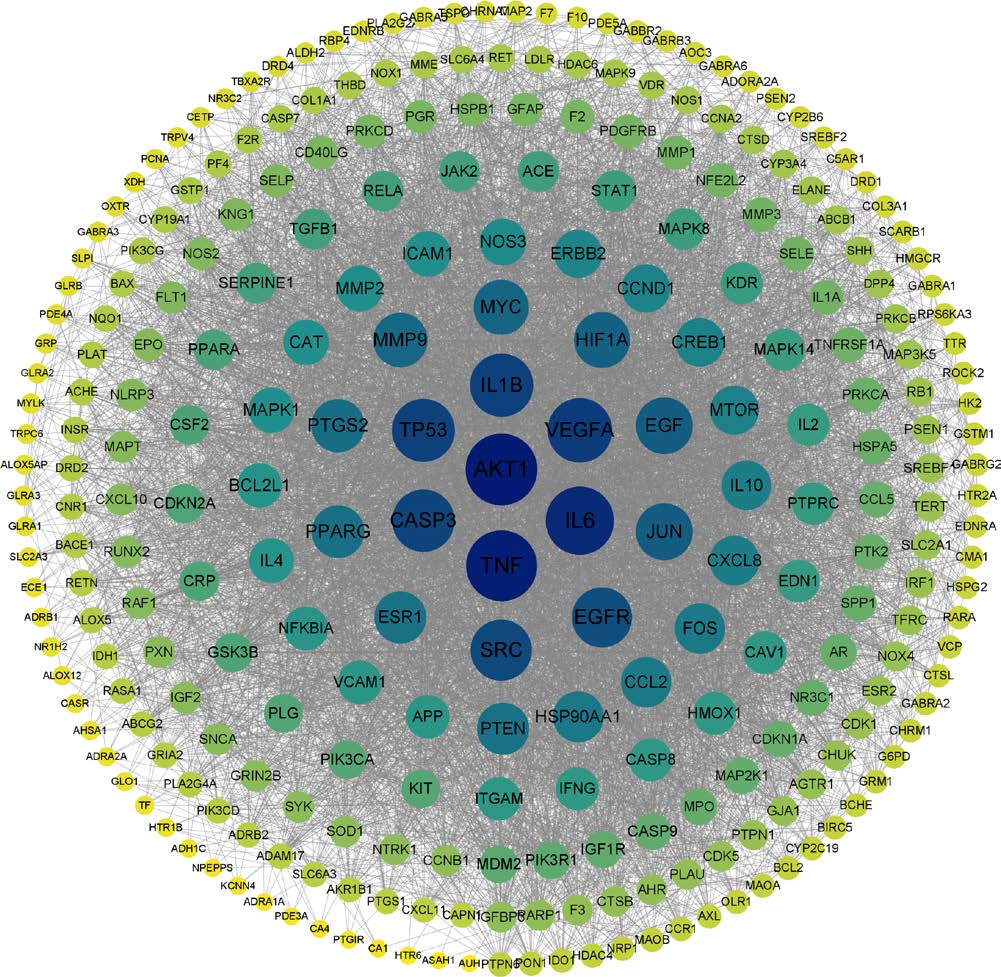
PPI network of potential targets (the size and color depth of nodes are proportional to the degree value). PPI = protein–protein interactional.

**Figure 5.**
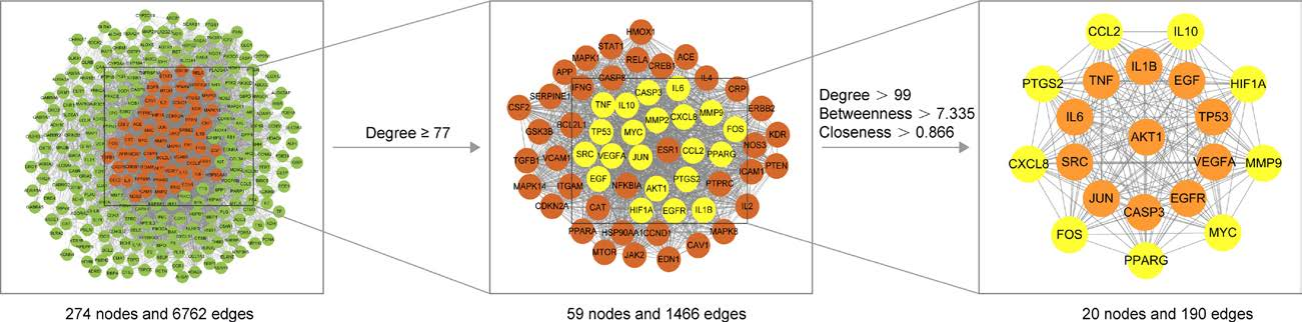
Key targets screening process. Among the selected key targets, the top 11 targets with the degree value are represented by orange and other targets are represented by yellow. The thickness and color depth of the edge are directly proportional to the correlation between the targets.

**Figure 6.**
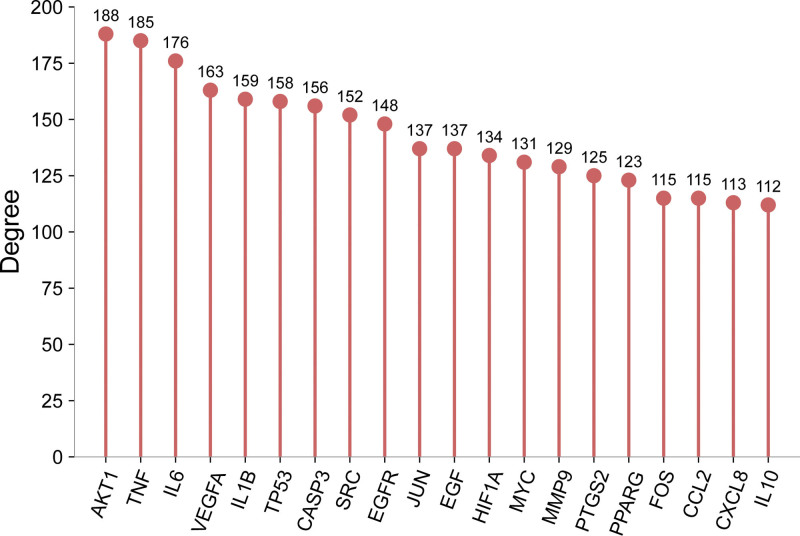
Point bar graph of degree-value points for 20 key targets.

All 274 potential targets were imported into the STRING database to obtain a PPI network. The results were imported into Cytoscape for network topology analysis and visualization, which showed that the PPI network contained 274 targets and 6762 protein interactions with an average degree value of 49.4, with the target color and size reflecting the degree value (Fig. 4). With a node degree value greater than or equal to 2 times the median (degree ≥ 77) as the first screening criterion, 59 candidate targets were obtained. The betweenness and closeness values of candidate targets were obtained by analysis of the cytoNCA plug-in. Targets larger than the median of degree, betweenness, and closeness values were screened, identifying 20 key targets (Fig. 5). Figure 6 shows the degree-value point bar graph of key targets and the top 11 targets of value were AKT1, TNF, IL6, VEGFA, IL1B, TP53, CASP3, SRC, EGFR, JUN, and EGFR, which we speculate may be involved in and play a key role in the pharmacological process of GBLs for VD. The 20 key targets were matched with the corresponding active ingredients and filtered by degree values. The top-ranked active ingredients were quercetin, luteolin, ginkgolide B, and kaempferol. This was consistent with the top targets obtained in Section 3.3, further suggesting that these ingredients may be the key ingredients for the treatment of VD by GBLs. Cluster analysis of the key target network using the MCODE plug-in revealed that the key targets were tightly connected and formed a cluster with high protein–protein correlation. The functions of these proteins were tightly linked and might interact to perform specific biological functions. It was further demonstrated that these proteins might play a significant role in the mechanism of VD treatment by GBLs.

### 3.5. Results of GO and KEGG enrichment analysis

**Figure 7.**
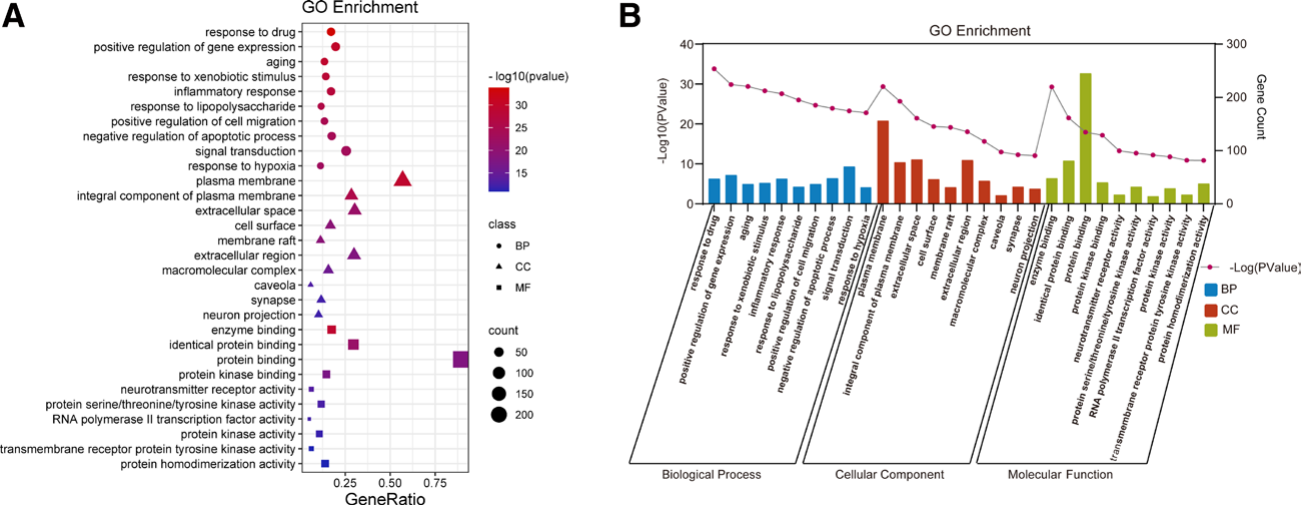
(A) Bubble diagram of GO enrichment analysis (the bubble size represents the number of genes enriched and the color represents the significance of enrichment); (B) GO enrichment analysis histogram. BP = biological processes, CC = cell composition, GO = gene ontology, MF = molecular functions.

To investigate the potential synergistic mechanism of GBLs for VD treatment, GO and KEGG analyses of potential targets were performed using the DAVID database, which contained 938 biological processes, 110 cellular compositions, 192 molecular functions, and 181 signaling pathways. Figure 7A and B shows the bubble diagram and histograms of the top 10 GO entries screened according to their *P* values. The results indicated that the biological processes mainly involved the response to drugs (GO:0042493), positive regulation of gene expression (GO:0010628), aging (GO:0007568), inflammatory response (GO:0006954), negative regulation of apoptotic process (GO:0043066), and response to hypoxia (GO:0001666). Cellular composition mainly involved the plasma membrane, extracellular space, cell surface, membrane raft, and synapse. Molecular functions mainly involved enzyme binding (GO:0019899), identical protein binding (GO:0042802), protein kinase binding (GO:0019901), neurotransmitter receptor activity (GO:0030594), and RNA polymerase II transcription factor activity (GO:0004879).

**Figure 8.**
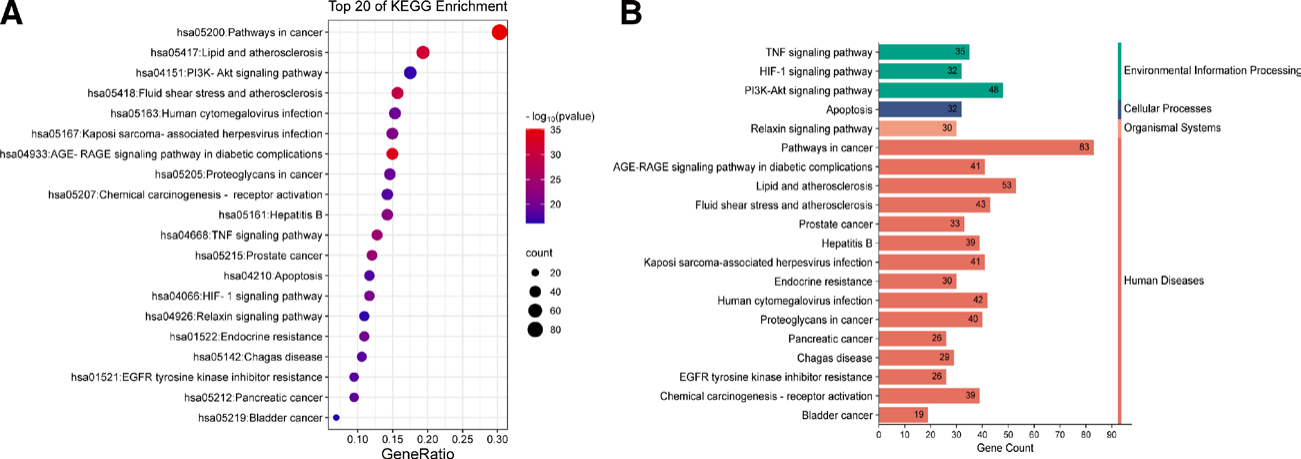
(A) Bubble diagram of KEGG pathway enrichment analysis; (B) KEGG pathway classification diagram. KEGG = Kyoto Encyclopedia of Genes and Genomes.

**Figure 9.**
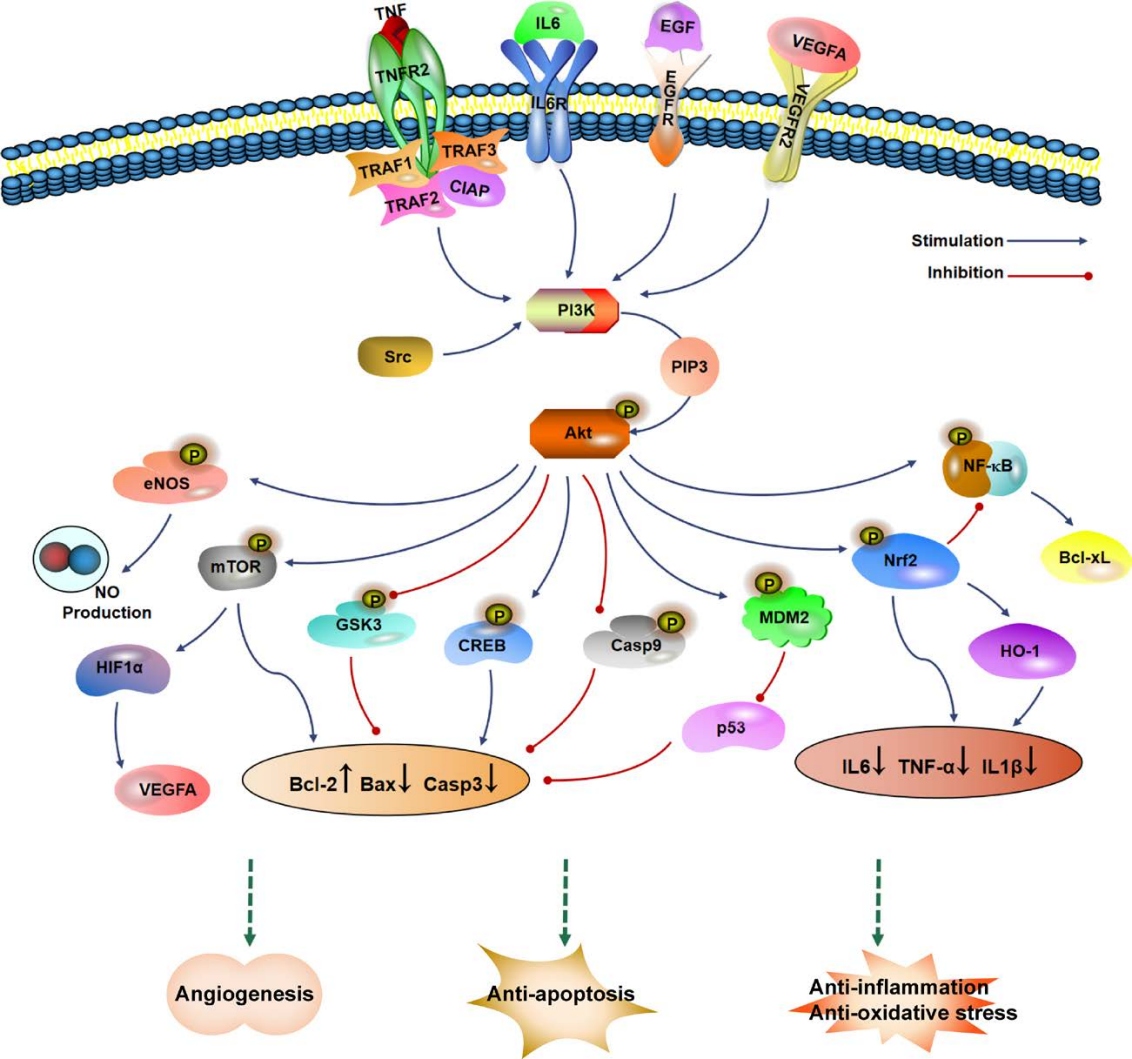
Predicted mechanism of potential targets of GBLs for VD treatment *via* the PI3K-Akt signaling pathway. GBLs = *Ginkgo biloba* L. leaves, VD = vascular dementia.

Figure 8A shows a bubble diagram of the top 20 KEGG signaling pathways screened according to *P* value, and Figure 8B ranks them according to the pathway category. Removing signaling pathways containing specific diseases, KEGG pathway analysis showed high target enrichment in lipid and atherosclerosis, the PI3K/Akt signaling pathway, fluid shear stress and atherosclerosis, the AGE–RAGE signaling pathway in diabetic complications, the TNF signaling pathway, the HIF-1 signaling pathway, apoptosis, and the endocrine resistance signaling pathway. Among them, the PI3K/Akt signaling pathway ranked second in terms of enrichment, involving a total of 48 potential targets, and is an important signaling pathway that regulates neuronal cell survival and modulates cognitive function in the brain,^[28]^ playing an important role in mechanisms related to learning and memory consolidation.^[29,30]^ The repair of various organs in the human body usually proceeds through the PI3K/Akt pathway, especially in the central nervous system.^[31]^ After being phosphorylated by upstream signals, the PI3K/Akt pathway can stimulate downstream proteins to induce a cascade response that is involved in neuroprotection by inhibiting apoptosis, alleviating inflammation and oxidative stress, stimulating angiogenesis, improving cerebral circulation, and many other biological processes (Fig. 9). This is closely related to the occurrence and development of VD and seems to be a key pathway for the treatment of VD by GBLs.

### 3.6. Analysis of molecular docking and visualization results

**Figure 10.**
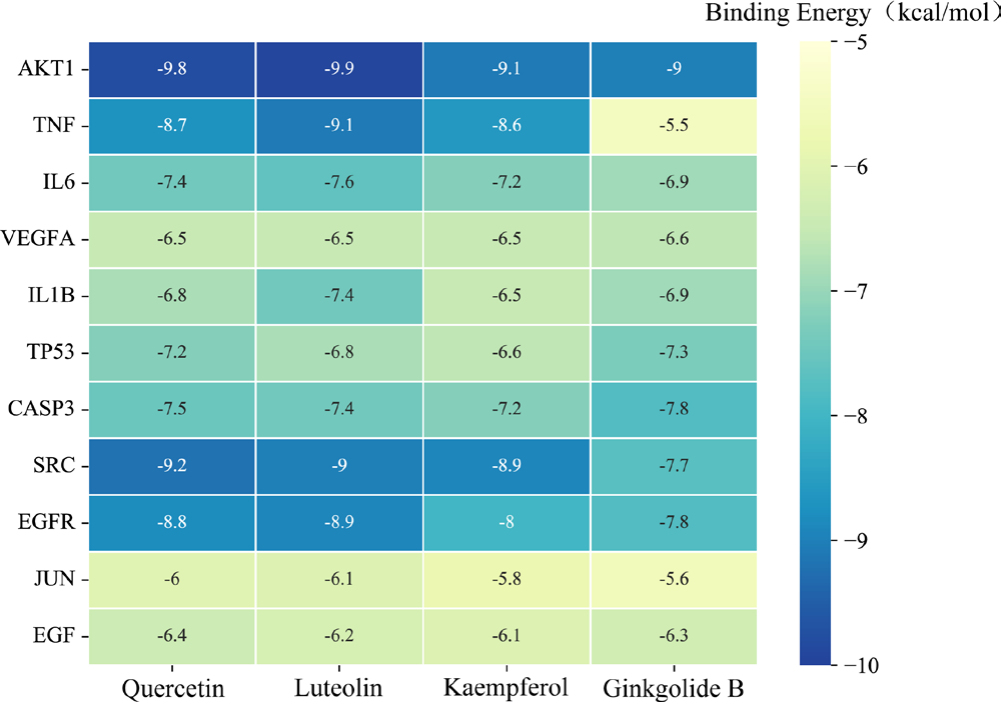
Molecular docking binding energy heatmap (the color shade is inversely proportional to the magnitude of the binding energy).

To further investigate the intrinsic molecular mechanisms of GBLs in VD treatment, molecular docking was performed using AutoDock Vina 1.1.2. The lower the binding energy of the ligand to the receptor, the more stable the binding interaction. Binding energies less than −5 kcal/mol indicate a good binding capacity, and less than −7 kcal/mol indicate a strong binding capacity.^[32]^ The docking binding energy heatmap showed that all 44 groups had docking binding energies less than −5.0 kcal/mol, and most of them had binding energies less than −6.0 kcal/mol, with an average binding energy of −7.43 kcal/mol. This suggested that all molecules have strong binding affinities (Fig. 10). The combinations with stronger binding interactions were AKT1 and luteolin, AKT1 and quercetin, and SRC and quercetin, with −9.9, −9.8, and −9.2 kcal/mol, respectively, indicating that these active components have strong binding affinity for their targets and may play an important role in the treatment of VD by GBLs.

**Figure 11.**
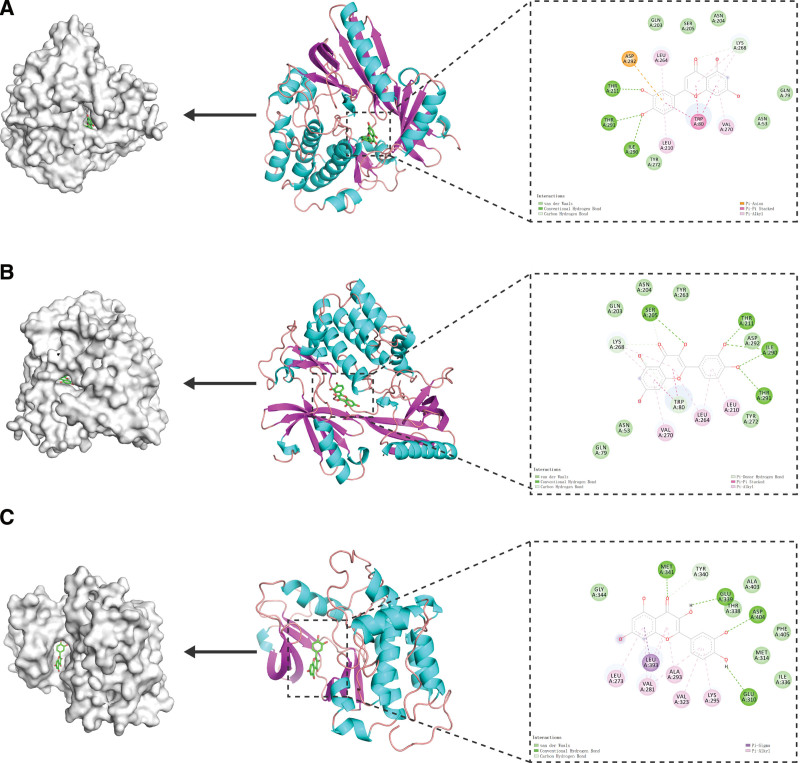
(A) AKT1–luteolin interaction surface and 2D and 3D images of the molecular docking results; (B) AKT1–quercetin interaction surface and 2D and 3D images of the molecular docking results; (C) SRC–quercetin interaction surface and 2D and 3D images of the molecular docking results.

The 3 groups of small molecules with the highest binding affinities were visualized docked to the target proteins (Fig. 11) and we found that all 3 groups of small molecules were bound in a deep cavity inside the receptor protein, and they had good shape complementarity. The binding sites were both hydrophobic and hydrophilic, involving various modes of interaction, such as hydrogen bonding, van der Waals forces, carbon–hydrogen bonding, and π–π stacking. Luteolin forms hydrophobic interactions with the amino acid residues Ile290, Trp80, Val270, and Tyr272 in the active site of AKT1. Additionally, luteolin can form a π–π stacking interaction with Trp80 to stabilize its binding and can also form hydrogen bonds with Thr291, Ile290, and Thr211 to contribute to the stability of the complex. AKT1 residues that form hydrophobic interactions with quercetin include Tyr263, Tyr272, Leu210, Leu264, Val270, and Trp80, in addition to π–π stacking interactions with Trp80 to stabilize its binding and hydrogen bonds with Ser205, Thr211, Ile290, and Thr291 to anchor the protein. Quercetin can form strong hydrophobic interactions with residues Leu393, Leu273, Val323, Met341, and Tyr340 of SRC and can also form hydrogen bonds with Glu339, Asp404, Glu310, and Met341 to stabilize binding.

### 3.7. The root mean square deviation (RMSD), root mean square fluctuation (RMSF), and radius of gyration (ROG) results in molecular dynamics simulation

**Figure 12.**
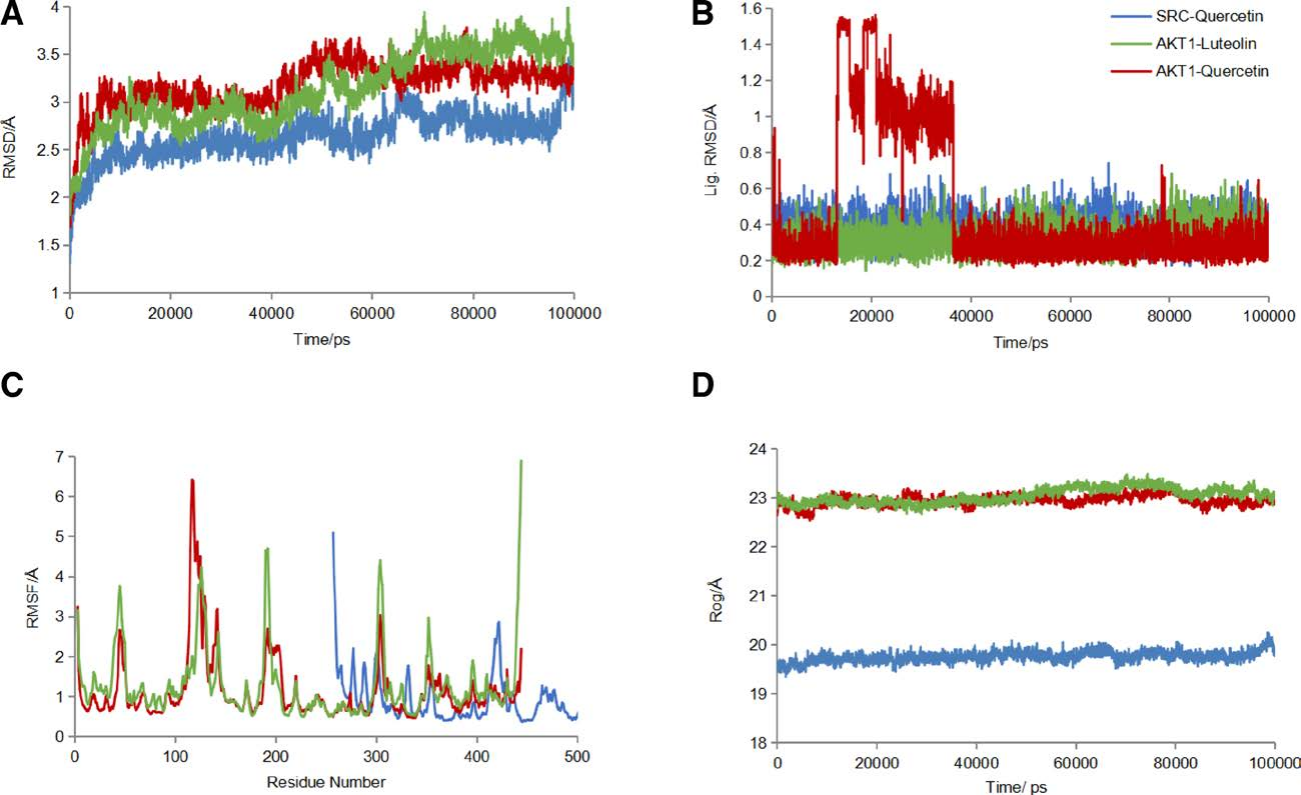
(A) RMSD curves of complexes during molecular dynamics simulations; (B) RMSD curves of small molecules during molecular dynamics simulations; (C) RMSF curves of amino acid residues during molecular dynamics simulations; (D) ROG curves of proteins during molecular dynamics simulations. RMSD = root mean square deviation, RMSF = root mean square fluctuation, ROG = radius of gyration (the green curve represents AKT1–luteolin, the red curve represents AKT1–quercetin, and the blue curve represents SRC–quercetin).

The RMSD curve indicates fluctuations in the protein conformation. Figure 12A shows that after 100 ns of the molecular dynamics simulation, AKT1-luteolin, AKT1-quercetin, and SRC-quercetin stabilized after 80, 50, and 60 ns, respectively. Figure 12B shows the RMSD fluctuation curves for the small molecules. The RSMD of small molecules of AKT1–luteolin and SRC–quercetin complexes tended to be smooth throughout the dynamics, and the RSMD of small molecule of AKT1–quercetin complex fluctuated more in the first 40 ns and tended to be smooth after 40 ns. This result indicates that the binding of small molecules to the receptor protein does not lead to a continuous and significant change in their conformation and that the combination of the 2 is relatively stable. The small molecule stably binds to form a tightly bound complex with the protein.

The RMSF curve indicates the fluctuation in amino acid residues in the protein. Figure 12C shows the molecular dynamics results of AKT1–luteolin, AKT1–quercetin, and SRC–quercetin, indicating that the intermediate regions of the proteins have greater residue flexibility than other regions and these regions are loop-based polypeptide chains and therefore have higher flexibility.

The ROG curve indicates the compactness of the overall protein structure. Figure 12D shows that AKT1–luteolin, AKT1–quercetin, and SRC–quercetin had stable gyration radii. This result is consistent with the RMSD results, which imply that the protein conformation is stable and tightly folded. Thus, it can be speculated that protein stability is unaffected by small molecule binding.

### 3.8. Molecular conformational changes before and after molecular dynamics

**Figure 13.**
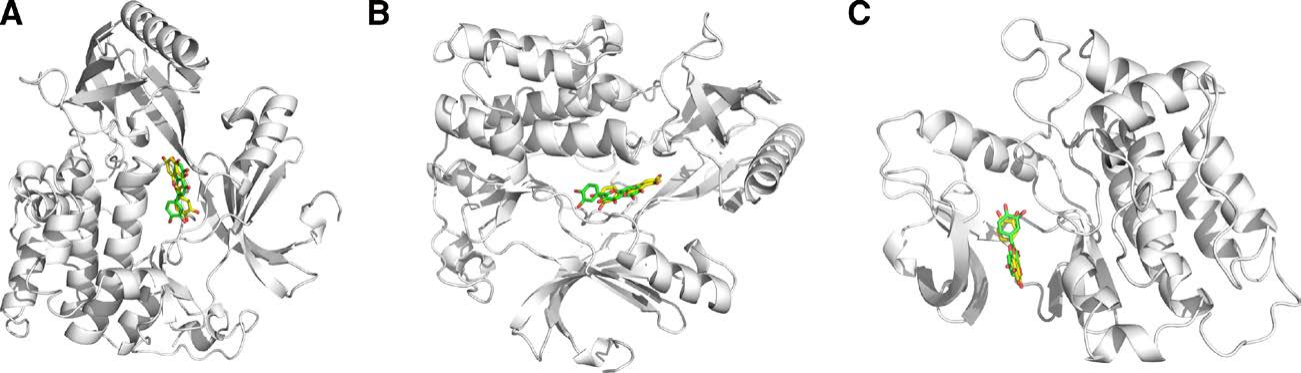
(A) Molecular conformational changes before and after AKT1–luteolin dynamics; (B) Molecular conformational changes before and after AKT1–quercetin dynamics; (C) Molecular conformational changes before and after SRC–quercetin dynamics.

Using the alignment command of PyMol software to compare the conformation of small molecules before and after molecular dynamics (Fig. 13), the green small molecule indicates the docked conformation and the yellow small molecule indicates the conformation after 100 ns of dynamics simulation. The 2 binding sites were highly consistent, and the dynamics did not shift the small-molecule binding sites significantly. Combined with the RMSD of the small molecules in Figure 12B, it is clear that the small molecules are stable in binding to the receptor protein.

### 3.9. Results of free energy calculations for binding and interacting residues during molecular dynamics simulations

Based on the MM/GBSA equation, the binding free energies between small molecules and proteins were calculated by taking 80–100 ns trajectories for AKT1–luteolin, 50–100 ns trajectories for AKT1–quercetin, and 60–100 ns trajectories for SRC–quercetin. The results showed that the binding free energies of AKT1–quercetin, AKT1–quercetin, and SRC–quercetin were −22, −20, and −34 kcal/mol, respectively. The van der Waals potential energy and electrostatic energy between the 3 groups of small molecules and proteins were favorable for the binding of the ligand and protein. Specific calculations of the binding free energies are presented in Table 3.

**Table 3:** The binding free energy and energy components of molecular dynamics simulation.

Energy component	AKT1–luteolin	AKT1–quercetin	SRC–quercetin
ΔE_vdw_	–35.4198 ± 2.9668	–29.9003 ± 3.9327	–34.3507 ± 2.7861
ΔE_elec_	–21.8734 ± 8.4251	–47.2561 ± 10.7459	–47.5331 ± 5.4971
ΔE_GB_	39.6523 ± 5.939	61.545 ± 7.6662	53.1521 ± 3.106
ΔG_Bind_	–22.257 ± 2.5877	–20.3277 ± 4.9706	–34.2975 ± 2.2925

ΔE_GB_ = polar solvation, ΔE_elec_ = electrostatic energy, ΔE_vdW_ = van der Waals energy, ΔG_Bind_ = the binding free energy.

## 4. Discussion

VD is a cerebrovascular disease characterized by severe cognitive and memory impairment. Apoptosis, inflammatory response, and oxidative stress due to cerebrovascular causes (especially CCH) are thought to be the molecular mechanisms closely associated with VD.^[6,33]^ In addition, mechanisms such as amyloid-β deposition, mitochondrial dysfunction, impaired blood–brain barrier (BBB), and neurotransmitter system dysfunction have also been suggested to be associated with VD. These pathological mechanisms are not independent and may interact with each other to produce a pathogenic cascade response. In this study, GO analysis revealed that potential targets were associated with biological processes, such as apoptosis, inflammatory response, cell migration, lipopolysaccharide response, hypoxic response, and aging, which are consistent with the pathological process of VD. In addition, this study identified quercetin, luteolin, kaempferol, and ginkgolide B as the main active ingredients of GBLs for the treatment of VD. The first 3 belong to the class flavonoids and the latter to diterpene lactones. They are all closely related to VD and play essential roles in neuroprotection in terms of anti-apoptosis, anti-inflammation, anti-oxidation, and promotion of blood circulation. Therefore, we believe that GBLs are promising anti-VD lead drugs for further development.

Quercetin has been widely used in cardio–cerebrovascular and neurodegenerative diseases because of its anti-inflammatory, antioxidant, anti-apoptotic, and neurogenesis-enhancing properties, which exert neuroprotective effects in various research models.^[34]^ It was found that quercetin enhanced neuronal resistance to oxidative stress and excitotoxicity by modulating cell death mechanisms, increasing the expression of longevity factors, and preventing neuroinflammation in the hippocampus by decreasing astrocyte markers and pro-inflammatory factor protein levels.^[35]^ In addition, quercetin plays a protective role in the apoptosis and degeneration of hippocampal neurons.^[36]^ Luteolin reduces brain reactive oxygen species (ROS) levels and modulates inflammatory factors through anti-oxidative stress, anti-inflammatory, and anti-apoptotic effects to achieve protective effects for neurons and inhibit or prevent neuroinflammation and cognitive decline caused by neuronal death.^[37]^ Kaempferol can bind to vascular endothelial growth factor (VEGF), enhance the angiogenic effects of VEGF in different models, and prevent vascular damage by alleviating oxidative stress and reducing inflammatory marker levels.^[38,39]^ Ginkgolide is a key constituent of GBE and the most studied one is ginkgolide B, which is recognized as a natural neuroprotective agent through its beneficial effects of reducing neuroinflammation, inhibiting neuronal apoptosis, increasing cerebral blood flow, and promoting learning and memory capacity in various neurological disease models.^[40,41]^

Although apoptosis, inflammation, and oxidative stress usually exhibit distinct mechanisms, they are usually involved in the molecular regulatory pathways of neuronal survival and death induced by vascular dysfunction. These molecular regulatory pathways are also activated in a sequential and interdependent manner. Similarly, the key targets obtained in this study were closely related to these 3 processes. Molecular docking and molecular dynamics simulations showed that the top 11 key targets, according to the degree value, had good binding affinity for the key active ingredients and the top 3 protein–small molecules had good binding stability to each other. Among these, AKT serine/threonine protein kinase 1 (AKT1), which has the strongest binding capacity to luteolin and quercetin, is the main target of GBLs and plays a key role in the inhibition of apoptosis.^[42]^ Neuronal apoptosis, especially in hippocampal neurons, is the main cause of symptoms of dementia. It has been shown that increased AKT1 phosphorylation can downregulate Caspase-3 (CASP3) activation, thereby increasing the survival of hippocampal neurons and reducing learning and memory impairment.^[43]^ AKT1 can be activated by extracellular signaling through a phosphatidylinositide 3-kinases (PI3K)-dependent mechanism, and activated AKT1 is involved in the cell survival pathway by inhibiting the apoptotic process, which is the core of the PI3K/Akt signaling pathway.^[44]^ Based on the results of KEGG analysis, the PI3K/Akt signaling pathway appears to be a critical pathway for the GBLs involved in VD. PI3K-activated AKT can mediate the inhibition of neuronal apoptosis and promote neuroprotection by phosphorylating multiple downstream target molecules, including GSK-3β, mTOR, CREB, Bcl-2 protein family, and CASP3. CASP3 is the most important protease in the apoptotic signaling pathway and is considered a key mediator of neuronal apoptosis.^[45]^ Considerable studies have shown that Casp3 expression is increased in the hippocampus of VD rats and that reducing apoptotic proteins in the hippocampus of VD rats can inhibit neuronal apoptosis, thereby alleviating the symptoms of VD and improving cognitive impairment.^[46,47]^ The Bax/Bcl-2 ratio is an important indicator of apoptosis,^[48]^ and a higher Bax/Bcl-2 ratio indicates a stronger pro-apoptotic capacity. According to immunohistochemical findings, VD rats had substantially higher levels of Bax expression and substantially lower levels of Bcl-2 expression, which raised the Bax/Bcl-2 ratio.^[46,47]^ It was found that GBE inhibited apoptosis of hippocampal neurons in mice after stroke by regulating the expression of Bax/Bcl-2 and Casp3.^[49]^ The PI3K/Akt signaling pathway could also downregulate the Bax/Bcl-2 ratio, alleviate apoptosis, and exert neuroprotective effects.^[50,51]^ Therefore, we hypothesized that GBLs might be involved in regulating neuronal apoptosis primarily through the PI3K/Akt pathway to mitigate the developmental process of VD.

Neuroinflammation is increasingly recognized as a risk factor for dementia. Numerous studies have demonstrated that inflammatory factors play a key role in the pathogenesis of VD and are important biomarkers for VD diagnosis.^[52]^ GO analysis revealed that molecular functions, such as enzyme binding, identical protein binding, general protein binding, and protein kinase binding, are involved in the overall neuroinflammatory response. Among the key targets, increased levels of the inflammatory cytokines interleukin-6 (IL6), tumor necrosis factor (TNF), and interleukin-1β (IL1B) all independently contribute to cognitive impairment. A recent meta-analysis showed that moderate to high elevations in blood levels of IL6 and TNF-α were associated with a diagnosis of VD and that for each unit increase in IL-6 levels, the risk of VD increased by 28%.^[53]^ TNF-α is a key mediator of neuroinflammation and can induce enhanced pro-apoptotic responses in combination with other pro-inflammatory cytokines in an inflammatory environment. It has also been found that TNF-α expression in the brains of patients with VD is highly correlated with p53 expression, and both of these proteins act together to initiate the neuronal apoptotic program, leading to neuronal death.^[54]^ IL1β enhances the activity of matrix metalloproteinase-9 (MMP9), which in turn causes BBB impairment, leading to significant vascular endothelial dysfunction, inflammation, and a more rapid decline in cognitive function.^[11]^ These pro-inflammatory cytokines can also contribute to the development of VD by activating microglia, damaging neurons through oxidative stress, directly damaging white matter, and inducing dysfunction of brain microvascular endothelial cells. Microglia are the major inflammatory cells in the central nervous system, and their mediated neuroinflammation plays an important role in VD.^[55]^ SRC kinase (SRC) plays a key role in triggering microglia activation and is a key factor involved in the development of neuroinflammation.^[56]^ In addition, both pro-inflammatory cytokines and vascular endothelial dysfunction can induce overexpression of vascular cell adhesion molecule 1 (VCAM1). Previous studies have confirmed that VCAM1 in brain endothelial cells can activate microglia, inhibit hippocampal neural progenitor cell activity, and impair cognitive function.^[57]^ Quercetin, a key ingredients of GBLs, can improve the function and energy metabolism of cerebrovascular endothelial cells, reduce neurovascular injury, and participate in the prevention and delay of neurodegenerative diseases by targeting VCAM1.^[58]^ Oxidative stress is considered one of the contributing factors in the pathogenesis of VD, where excessive production of ROS in the brain and imbalance with cellular antioxidant systems eventually leads to cellular damage, including mitochondrial damage and neuronal apoptosis.^[59,60]^ GO analysis involves biological processes such as the inflammatory response, response to lipopolysaccharides, response to hypoxia, and aging, all of which are closely related to oxidative stress and inflammation. Sustained oxidative stress can activate chronic inflammatory mechanisms and inflammation increases ROS production, which in turn induces oxidative stress and further exacerbates the inflammatory response, resulting in a vicious oxidative stress–inflammation cycle that can lead to cell death in severe cases.^[61,62]^ The PI3K/Akt pathway regulates downstream targets that play a role in neuroinflammation and oxidative stress, and the potential target NFE2L2 encoding the nuclear factor erythroid two-related factor (Nrf2) is a major regulator against inflammation and oxidative stress. It was found that activation of the Nrf2/heme oxygenase 1 (HO-1) pathway downregulated the production and release of pro-inflammatory mediators in microglia and subsequently protected neurons from inflammatory damage, and that upregulation of Nrf2 and HO-1 was reliant on activation of the PI3K/Akt signaling pathway.^[63]^ In addition, quercetin was found to reduce lipopolysaccharide-induced ROS production and inhibit inflammatory responses through the ROS-regulated PI3K/Akt/NF-κB signaling pathway, exerting a protective effect in atherosclerosis models.^[64]^

Neurorepair of VD is closely related to angiogenesis, which has been shown to mitigate the reduction in cerebral blood flow caused by different types of brain injury and to prevent and delay the occurrence of VD.^[65]^ After the onset of VD, cerebral microangiogenesis can promote neurological recovery by improving cerebral microcirculation and the blood oxygen content of surrounding tissues, thus improving learning and memory capacity.^[66,67]^ Vascular endothelial growth factor (VEGF) is by far the most significant and specific pro-vascular growth regulator.^[68]^ In addition to its classical effects, such as angiogenesis and increased vascular permeability, VEGF can also exert neurotrophic, neuroprotective, and neurogenic activity by binding to its receptor VEGFR2.^[69]^ It was found that VEGF can activate the downstream PI3K/Akt pathway, which is involved in regulating endothelial cell differentiation and angiogenesis to repair brain tissue and neurological damage.^[70,71]^ In addition, the epidermal growth factor receptor (EGFR) can activate the PI3K/Akt/mTOR pathway and increase VEGF secretion.^[72]^ Endothelial nitric oxide synthase (eNOS) deficiency leads to endothelial dysfunction. This results in severe brain damage and cognitive deficits.^[73,74]^ Akt1 is the major Akt isoform that regulates normal physiological functions of endothelial cells and preferentially regulates the phosphorylation of eNOS.^[75,76]^ VEGF stimulates Akt-mediated eNOS phosphorylation and promotes nitric oxide (NO) release thereby participating in the regulation of vascular tension, angiogenesis, vascular remodeling, and maintenance of vascular homeostasis.^[77,78]^ SRC regulates vascular endothelial cell proliferation and differentiation and is involved in growth factor-induced (including VEGF) vascular permeability, BBB destruction, and cerebral angiogenesis after ischemic stroke.^[79,80]^ The PI3K/Akt pathway is involved in regulating the expression of angiogenic factors such as NO and angiopoietin.^[81]^ In addition, SRC mediates the PI3K/Akt signaling pathway to stimulate eNOS phosphorylation,^[82,83]^ and eNOS catalyzes the synthesis of low levels of NO, thereby protecting the brain tissue.

## 5. Conclusion

In this study, the molecular mechanisms of GBLs in VD treatment were investigated using network pharmacology, molecular docking, and molecular dynamics simulations. Quercetin, luteolin, kaempferol, and ginkgolide B are the key active ingredients of GBLs against VD and act on multiple signaling pathways, such as PI3K/Akt, lipid and atherosclerosis, fluid shear stress, and atherosclerosis through the regulation of AKT1, TNF, IL6, VEGFA, IL1B, and other targets, among which the PI3K/Akt signaling pathway seems to play a key role. These findings suggest that GBLs play a neuroprotective role by reducing apoptosis, alleviating inflammation, limiting oxidative stress, and promoting angiogenesis through multi-ingredient, multi-target, and multi-pathway interactions involved in the treatment of VD, which is generally consistent with the therapeutic pathways of VD reported in previous studies. Molecular docking and molecular dynamics simulations suggested that the interactions between AKT1–luteolin, AKT1–quercetin, and SRC–quercetin may be an important link in this mechanism. GBLs may serve as promising complementary and alternative drugs against VD; however, in vivo*/*in vitro experiments are still needed to further validate their mechanism of action. This study comprehensively elucidates the underlying pharmacological mechanism of GBLs in the treatment of VD and lays the foundation for further optimization of experimental design to obtain more reliable results.

## Author contributions

**Conceptualization:** Jienuo Pan, Jiqin Tang.

**Data curation:** Jienuo Pan, Jiqin Tang, Jialin Gai.

**Formal analysis:** Jienuo Pan, Yilan Jin, Bingshun Tang.

**Funding acquisition:** Jiqin Tang.

**Methodology:** Jienuo Pan, Yilan Jin, Xiaohua Fan.

**Software:** Jienuo Pan, Jiqin Tang.

**Supervision:** Jiqin Tang, Xiaohua Fan.

**Visualization:** Jienuo Pan, Jialin Gai, Bingshun Tang.

**Writing – original draft:** Jienuo Pan, Jiqin Tang, Yilan Jin.

**Writing – review & editing:** Jienuo Pan, Jiqin Tang, Jialin Gai, Yilan Jin, Bingshun Tang, Xiaohua Fan.
